# The Structural Basis of Peptide Binding at Class A G Protein-Coupled Receptors

**DOI:** 10.3390/molecules27010210

**Published:** 2021-12-30

**Authors:** Oanh Vu, Brian Joseph Bender, Lisa Pankewitz, Daniel Huster, Annette G. Beck-Sickinger, Jens Meiler

**Affiliations:** 1 Deparment of Chemistry, Vanderbilt University, Nashville, TN 37235, USA; oanh.t.vu.2@vanderbilt.edu; 2 Center for Structural Biology, Vanderbilt University, Nashville, TN 37232, USA; brian.bender@ucsf.edu (B.J.B.); lisa@simula.no (L.P.); 3 Department of Pharmacology, Vanderbilt University, Nashville, TN 37232, USA; 4 Institute for Medical Physics and Biophysics, Medical Department, Leipzig University, Härtelstr. 16–18, D-04107 Leipzig, Germany; daniel.huster@medizin.uni-leipzig.de; 5 Faculty of Life Sciences, Institute of Biochemistry, Leipzig University, Brüderstr. 34, D-04103 Leipzig, Germany; abeck-sickinger@uni-leipzig.de; 6 Leipzig University Medical Center, Institute for Drug Discovery, Departments of Chemistry and Computer Science, Leipzig University, Brüderstr. 34, D-04103 Leipzig, Germany

**Keywords:** peptide GPCR, class A GPCR, peptide docking, non-canonical amino acids

## Abstract

G protein-coupled receptors (GPCRs) represent the largest membrane protein family and a significant target class for therapeutics. Receptors from GPCRs’ largest class, class A, influence virtually every aspect of human physiology. About 45% of the members of this family endogenously bind flexible peptides or peptides segments within larger protein ligands. While many of these peptides have been structurally characterized in their solution state, the few studies of peptides in their receptor-bound state suggest that these peptides interact with a shared set of residues and undergo significant conformational changes. For the purpose of understanding binding dynamics and the development of peptidomimetic drug compounds, further studies should investigate the peptide ligands that are complexed to their cognate receptor.

## 1. Introduction

### 1.1. G Protein-Coupled Receptors Are a Significant Target of Therapeutic Intervention

With more than 800 members, G protein-coupled receptors (GPCRs) are the largest family of human transmembrane proteins [1]. They are key players in many physiological functions, regulate the majority of cellular processes, and are involved in numerous disease pathologies [2]. By subtracting the olfactory/odorant GPCRs involved in recognizing smells, about 400 human GPCRs are considered as druggable. Their substantial involvement in cellular signaling has established GPCRs as highly relevant pharmacological drug targets. About 34% of all drugs approved by the US Food and Drug Administration (FDA) achieve their therapeutic effects through GPCRs [3]. 

### 1.2. Peptide-Activated Receptors Are a Large Percentage of the GPCR Class A

Out of four classes of GPCR—A, B, C or F—Class A is the largest and most diverse group in humans. This subfamily has been investigated most extensively in drug discovery due to their available structural and experimental data. They conform with the common GPCR structural fold, such as a seven-transmembrane (7TM) helices domain, three extracellular loops, and three intracellular loops with ligand-binding pockets and a G-protein-binding region located in the extracellular and intracellular ends of the helix bundle, respectively [4]. The variety of drugs targeting GPCRs reflect the diversity of chemical signals that can be transduced by GPCRs, including small molecules, lipids, ions, and proteins [5,6]. In particular, according to the data from the GPCRdb server [4], the peptide- and protein-activated receptors are found to account for about 46% of all class A GPCRs in humans. For this review, we consider GPCRs that recognize classical peptides and peptide-like segments within larger protein domains and belong to the same category of receptors. Peptide-activated receptors are found across all rhodopsin-like subfamilies (α, β, γ, and δ) and the entire secretin family [7]. Given this coverage, it is unsurprising that many of the aforementioned blockbuster drugs (e.g., olmesartan, buserelin, and valsartan) target members of this receptor group. While Olmesartan and Valsartan serve as an angiotensin II receptor blocker (ARB) in treating hypertension [8,9], buserelin, a luteinizing hormone—releasing the hormone (LHRH) agonist, can be used to treat hormone responsive cancers, such as prostate and breast cancer [10]. In 2020, nearly 50 GPCR peptide drugs have been approved [11]. In accordance with this importance for therapeutic development, a full understanding of the structural and dynamical determinants of signaling for these molecules is necessary. This review covers what is known regarding these receptors structurally using various biophysical techniques and provides suggestions for future discovery routes.

### 1.3. Diversity of Peptide Ligands

Peptide ligands come in a variety of lengths and structures, although they share the common theme that they are ribosomally translated. Often, these peptide ligands are produced as pre-hormones that are subsequently processed to their active form. As a result, peptide ligands range in size from three amino acids (e.g., thyrotropin-releasing hormone (TRH)) up to ~100 amino acids (e.g., chemokine ligand 23 (CCL23)). In addition to size differences, many peptide hormones undergo post-translational modifications. Some of these modifications are necessary to increase the peptide half-life by inhibiting exopeptidases, such as N-terminal pyroglutamation (e.g., TRH and luteinizing hormone (LH) [12]) and C-terminal amidation (e.g., neuropeptide Y (NPY), pancreatic polypeptide (PP), and peptide YY (PYY) [13]). However, in some cases, these modifications serve dual purposes by acting as molecular recognition sites in their cognate receptors [14]. Other types of post-translational modifications include lipidation, bromination, and disulfide bridge formation. A summary of modifications is found in Table 1. These modifications further increase the diversity of chemical space available to peptide hormones beyond the canonical 20 amino acids. The size, sequence, shape, charge, structural dynamics, and chemical diversity allow for a vast degree of specificity between peptide hormones and their receptors. Furthermore, it is common for a given peptide hormone to exist in multiple isoforms, such as the neuropeptide Y (NPY) family, which consists of NPY, peptide YY (PYY), and pancreatic polypeptide (PP) and the endothelin peptides ET-1, ET-2, and ET-3. 

### 1.4. Reducing the Flexibility of Peptide Ligands Is Crucial for Success in Co-Crystallization

A significant challenge for the interpretation of structures determined via crystallization of peptide-activated receptors in complex with their cognate peptide ligand is the peptides’ inherent flexibility. Typically, small molecule antagonists and agonists will adopt a single conformation when interacting with a receptor and are fully encased in the receptor-binding pocket. Peptide ligands may adopt a single conformation in the binding pocket. However, due to their length, the remainder of the ligand can remain outside the binding pocket and be flexible. This conformation change is likely the reason that neurotensin 1 receptor (NTS1R) was crystallized with only residues 8–13 of the peptide, since residues 1–7 are expected to extend above the receptor pocket and remain unconstrained [25,26]. The peptide ligand of the apelin receptor, while full-length, was modified to incorporate a lactam ring, which significantly constrained the peptide’s flexibility [27]. Full-length chemokine crystallization is possible, as the portion of the chemokine that extends out of the binding pocket folds into a well-defined structural domain. However, the N-terminus of the receptor, known to recruit and bind the chemokines, has yet to be determined experimentally in its entirety [28,29,30]. 

### 1.5. Complexity of Peptide Ligand and Receptor Interactions

In addition, as was recently classified, many peptide ligands target multiple receptors adding to their signaling complexity [2]. This complex selectivity of peptide ligand/receptor interactions results in the peptide ligand biology’s common theme: Multi-ligand/multi-receptor systems. To date, evidence shows that the related ligands binding to the same receptor or the same ligand binding to two different receptors can adopt different bound state conformations and sustain deviating interaction networks [31,32], activating the receptors by the induced-fit or conformational selection. However, the system of multi-ligand/multi-receptor binding is different from the promiscuous binding of major histocompatibility complex (MHC) molecules to antigenic peptides. The GPCR-peptide binding relies on the conservation of residue pairwise interactions among evolutionarily related peptides and GPCRs receptors. In contrast, MHCs, due to their conformational flexibility, can fold into multiple active states to bind to a diverse set of antigenic peptides. A precedent kinetic study observed a slow rate, suggesting that the mutual configurational complementarity took time to be sufficient for flexible MHC and flexible peptides in order to form an initial complex [33]. This theme of multi-ligand/multi-receptor systems complicates the formulation of overarching binding and activation mechanisms that holistically explain this category of receptors, unlike what is known regarding receptors activated by bioamines [34,35,36]. Moreover, it complicates the development of selective probes and therapeutic agents. Therefore, it is critical for a full understanding of receptor/hormone biology to study each peptide ligand/receptor combination in detail before attempting to formulate generalizations that can be used for future drug development. This task is fundamental through many ongoing efforts in order to achieve this step. 

## 2. Comparison of Peptide Binding Modes across Class A GPCRs

### 2.1. Diversity in the Binding Modes of the Peptide Ligands to Class A GPCRs

The first crystal structure of a peptide-activated receptor was the CXCR4 receptor in 2010 [37]. The receptor structure was determined in the inactive state bound to both a small molecule antagonist and a peptidomimetic. This receptor structure was similar to what had previously been seen for aminergic [38,39] and nucleotide [40] receptors. However, an interesting difference was the presence of an β-hairpin in extracellular loop 2 (ECL2), a motif that has been present in all peptide-activated receptor structures reported since that time [41]. 

Moreover, two additional years passed before another peptide-activated receptor structure was determined. The year 2012 was a watershed year for this family with the structure determination of all four opioid receptor (OR) members (δOR [42], κOR [43], µOR [44], and NOP [45]), the protease-activated receptor type 1 (PAR1) [46], and the neurotensin type 1 receptor (NTS1R) [15]. Notably, the NTS1R structure was the first structure that was determined as a peptide-activated receptor in complex with its endogenous peptide ligand. Interestingly, NT’s binding depth was not as pronounced as seen for the aminergic and nucleotide ligands, suggesting that peptide ligands bind more superficially and predominantly interact with the extracellular loops. As the extracellular loops are the most divergent region of GPCRs, this prevented the extrapolation of this binding mode to other peptide ligands. 

Since 2012, additional peptide-activated receptor structures were determined. These included further chemokine receptors (CCR2 [47], CCR5 [48], CCR9 [49], and the viral US28 chemokine receptor [18]), both subtypes of the orexin [50,51] and angiotensin [52,53] receptors, the PAR2 receptor [54], the endothelin-B receptor [55], the neuropeptide Y type 1 receptor [56], the neurokinin 1 receptor [57], and the C5a receptor [58]. The binding pockets of peptide-activated GPCRs are uniformly wide due to the structured ECL2 but display a variety of hydrophobic and electrostatic conditions [42]. Of note, only a small subset of these structures has been determined with a peptide ligand bound. These include the chemokine receptors US28, CCR5, and CXCR4 [18,19,59,60], the endothelin-B receptor [55], the apelin receptor [17], the µ opioid receptor [61], the angiotensin type II receptor [53], and the C5a receptor [62].

**Figure 1:**
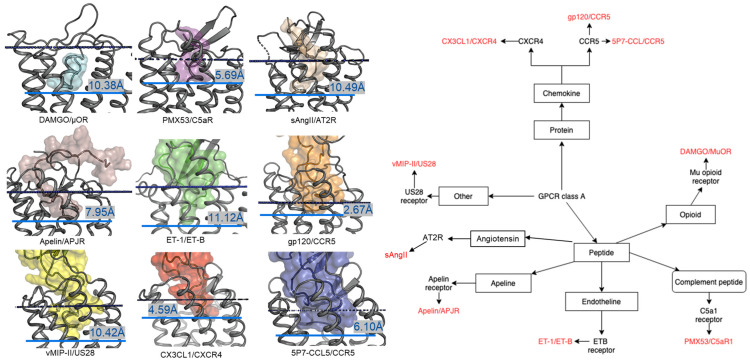
Overview of nine co-crystal structures of class A peptide-GPCR. (**Left**) Comparison of peptide binding modes and crystallized peptides DAMGO (cyan), PMX53 (magenta), sAngII (beige), apelin derivative (salmon), ET-1 (green), gp120 (orange), vMIP-II (yellow), CX3CL1 (red), and 5P7-CCL5 (blue) at the receptors µ opioid receptor (µOR) (PDB ID: 6DDE), complement component 5a receptor (C5aR) (PDB ID: 6C1R), angiotensin II type 2 receptor (AT2R) (PDB ID: 5XJM), apelin receptor (APJR) (PDB ID: 5VBL), endothelin B receptor (ET-B) (PDB ID: 5GLH), C-C chemokine receptor type 5 (CCR5) (PDB ID: 6MEO), US28 (PDB ID: 4XT1), CXC-chemokine receptor 4 (CXCR4) (PDB ID: 4RWS), and CCR5 (PDB ID: 5UIW), respectively [22,32,34,37,38,39,40,41,42]. All receptors were aligned in the transmembrane region. The approximated extracellular border of the transmembrane region is marked in the upper dotted dark blue lines. The membrane region of GPCR receptors was calculated using the PPM server [65]. The lower blue bars and texts illustrate the depth of penetration for each peptide ligand. (**Right**) Classification tree of eight class A GPCRs with their nine peptide ligands in those nine listed structures.

**Figure 2:**
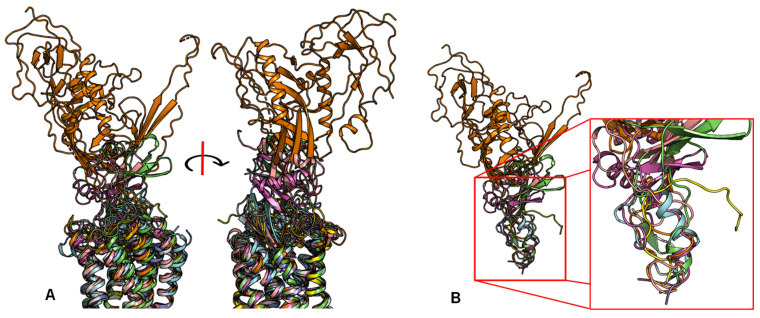
Despite the diversity in the peptide engagement, their overlapping region at the core of their binding pocket suggests common ligand-GPCR interactions. (**A**) Superimposition of the nine peptides/class A GPCR complexes. (**B**) Overlay of all peptide ligands and zoom-in of the peptide region at the cores of GPCRs.

In contrast to the observed orientation of NT(8-13), these ligands’ binding modes are very diverse, as seen in Figure 1. Peptide ligands can unwind their helix and adopt unstructured conformations to penetrate deep in the helical bundle via their N- or C-terminus, such as apelin. This observation of great diversity in peptide binding modes among GPCRs was also confirmed by the previous review work [63]. They can bind with both termini folded into the binding pocket, such as ET-1 or in a horseshoe manner, presenting a curved surface to the receptor, such as gp120. The ligands can bind deeply (sAngII, DAMGO, ET-1, and vMIP-II) or closer to the surface (gp120, CX3CL1, PMX53, and 5P7-CCL5). However, conservation in the peptide engagement mechanism among class A GPCRs has been investigated by combining earlier SAR studies and the alignment of interacting residues from recent GPCR-peptide structures. The authors suggested that common patterns in peptide-GPCR interactions were divided into four groups, depending on whether the peptide is cyclic or not and whether the GPCR interacts with the N- or the C-terminus of the peptide [64]. By superimposing the structures of the complexes, a common observation between the binding modes of different peptide ligands is that they often bind over an extended surface of the receptor (Figure 2A). More interestingly, we notice that peptides align surprisingly well at the core of the binding pocket (Figure 2B). Together with the conserved β-hairpin in ECL2, these observations suggest potential general themes conserved within GPCRs binding peptide-ligands. 

### 2.2. Peptide Ligands Affect the Conformation of the Extracellular Surface

**Figure 3:**
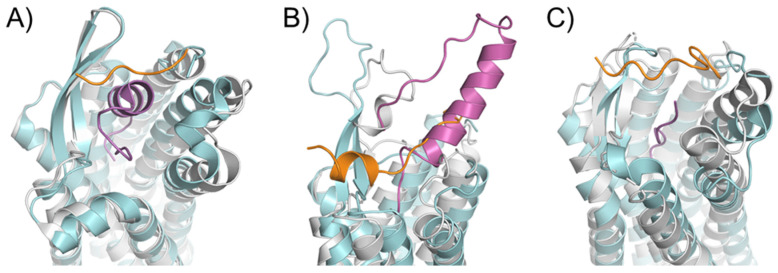
Rearrangements in the extracellular domain of peptide-activated GPCRs for peptide binding. (**A**) In the apo ET-B receptor (grey, PDB ID 5GLI), the N-terminus (orange) is lying over the ligand binding pocket. In the ET-1-bound state (cyan, PDB ID 5GLH), the bound ET-1 ligand (magenta) occupies the space of the N-terminus leading to its displacement [55]. (**B**) The crystal structure of antagonist-bound Y_1_ receptor (grey, PDB ID 5ZBQ) is also found with the N-terminus (orange) lying over the ligand binding pocket. The modeled peptide-bound Y1R (cyan) places the NPY ligand (magenta) in this space displacing the N-terminus [56]. (**C**) In the antagonist bound AT1 receptor (grey), the N-terminus (orange) extends over the pocket towards ECL2 [66]. In the AT2 receptor (cyan) bound to sAngII (magenta), the peptide binds deep within the pocket and the N-terminus lies over ECL3 [53].

An essential consequence of the extended binding surface area of peptide ligands is that their presence affects not only the deep binding pocket, but also the extracellular loops. This link between ligand engagement and GPCR loop conformation was recently demonstrated by the endothelin receptor structures [55]. This receptor was crystallized in the *apo* state and in complex with a peptide ligand. Interestingly, there was an extensive rearrangement of the extracellular domain in the peptide ligand presence (Figure 3A). This conformation rearrangement is expected to be the case for many peptide-activated receptor structures. In particular, the structural model of the Y_1_ receptor in complex with a small ligand found the N-terminus of the receptor lying over the binding pocket [56]. Mutagenesis studies confirmed that this portion of the receptor did not affect the binding properties of the small molecule or endogenous peptide. It was implied that the N-terminus needed to be displaced from this crystallized orientation to allow for the binding of the considerably larger NPY ligand (Figure 3B). This implication was modeled and presented with the crystal structure with an extensive use of orthogonal biophysical techniques, including NMR, cross-linking mass spectrometry, and mutagenesis. Additionally, the structure of the AT1R with a small molecule antagonist found the N-terminus lying over the ligand-binding pocket. In contrast, the AT2R structure, which was determined in the presence of a peptide analog sAngII, required the N-terminus to shift to allow for the access of the ligand to the orthosteric pocket (Figure 3C). 

### 2.3. ECL1 and ECL2 Bound Conformation Have Conversed across Class A Peptide-GPCRs 

**Figure 4:**
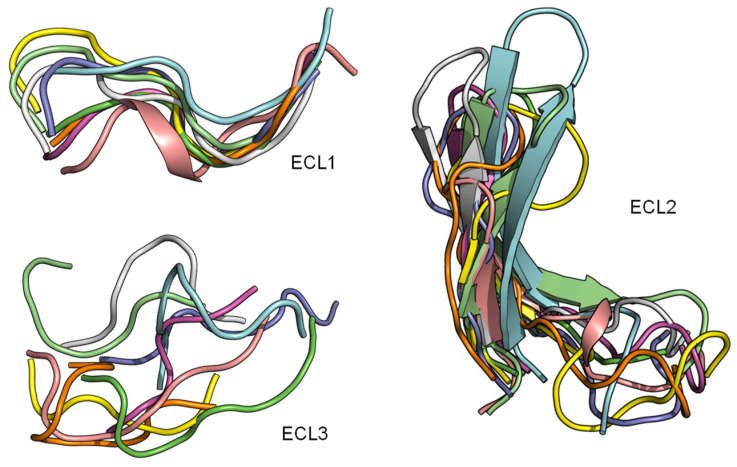
ECL1 and ECL2 have a conserved bound conformation compared to ECL3. Overlay of three extracellular loops.

The superimposition of the three extracellular loops of peptide GPCR class A shows that the bound conformations of ECL1 and ECL2 are considerably more conserved than the ECL3 (Figure 4). This observation sugsgests that the first two extracellular loops could support a general interface for peptide binding. Together with the conserved β-hairpin in ECL2, details of the ECL local structure and orientation are critical for recognizing specific peptide ligands. 

**Figure 5:**
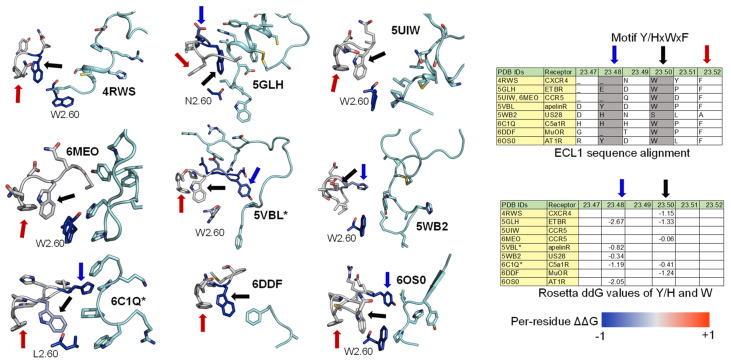
ECL1 and the role of motif Y/HxWxF in peptide binding among class A GPCRs with peptide ligands. (**Left**) Interactions among ECL1, residue 2.60, and the peptide. The interacting peptide residues are colored in cyan. Residues on ECL1 and 2.60 are colored based on their computed per-residue ΔΔG values (blue: Negative ΔΔG, darkest blue: −1 or below; grey: ΔΔG value of 0 or no interactions; red: Positive ΔΔG, darkest red: 1 and above). (**Right**) The tables show the sequence alignment of ECL1 and the three key residues in ECL1 motif Y/HxWxF are Y/H, W23.50, and F23.52, which are marked with blue, black, and red arrows, respectively.

Among the nine class A GPCR structures that we investigated, four ECL1s have a common motif Y/HxWxF, and eight of them possess an xWxF motif. This motif, together with residue 2.60, interacts favorably with the bound conformation of the peptides. More specifically, the aromatic Y/H sidechain tends to form hydrogen bonds or hydrophobic interactions with the adjacent peptide sidechain or backbones. Moreover, the residue F23.52 forms an π-π interaction to stabilize the conformation of W23.50, while W23.50 interacts with the peptide directly through hydrophobic interactions or indirectly through an π-π interaction with the nearby W/L2.60 residue (Figure 5-Left). Moreover, we quantified the strength of the aforementioned interactions by computing the per-residue ΔΔG with Rosetta on the contacting GPCR residues. A ΔΔG score is defined as the sum of the “two body” interaction scoring terms between each GPCR and peptide residues. We used the Rosetta Energy Function 2015 or REF2015, which encompasses a mix of weighted physics-based and knowledge-based scoring terms that were designed to evaluate the biomolecular structure, stability, and association. A previous publication has described the mathematical models and physical concepts that underlie the latest Rosetta energy function [67]. Supplementary Table S1 lists the two-body scoring terms that were included in the calculation of ΔΔG scores. The ΔΔG values of the three key residues (Y/H, W, and F) of the Y/HxWxF motif, together with the residue 2.60, are indicated by the colors on the images and reported in the table in Figure 5. In general, the ΔΔG values for those residues are negative, suggesting a favorable interaction energy. This quantitative analysis further confirms our observations regarding the common ECL1 mode of peptide engagement among the nine class A GPCR structures. 

**Figure 6:**
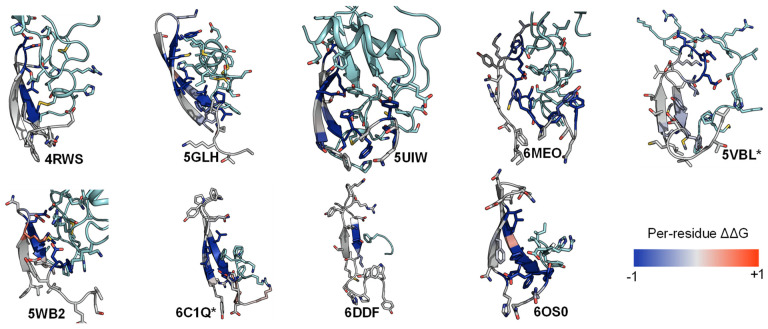
ECL2 β-hairpin and conserved residues interact with peptides of nine peptide/class A GPCR crystal structures.

**Figure 7:**
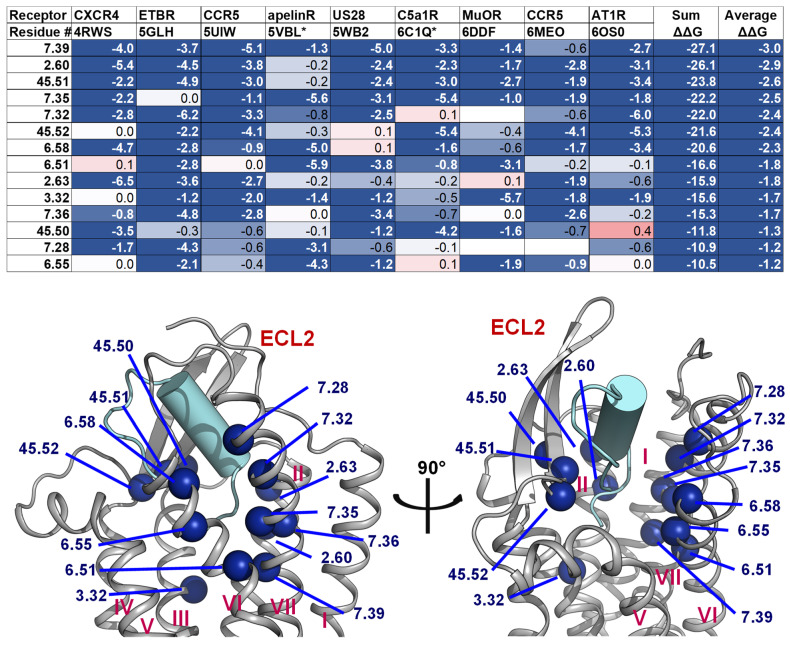
Residues with the strongest interactions according to the average computed ΔΔG suggest the common binding pocket of peptide ligands. (**Upper**) A table shows a list of residues with the top average computed ΔΔG values. The residues are numbered according on the Ballesteros-Weinstein numbering scheme [69]. For each residue position, the ΔΔG values are colored in the scale from −1 and less (blue) to 0 (white) to 1 and above (red). The absence of the ΔΔG values indicates that the corresponding residues do not interact with the peptide ligands. Two final columns of the table contain the sum and the average ΔΔG values across nine peptide-class A GPCR structures, respectively. The residue list is sorted in their ascending average ΔΔG order. (**Lower**) Front and side view of the common peptide binding pocket towards the core of nine class A GPCR structures. The top residues in the upper table are mapped on the ET-1/ETB structure (PDB ID: 5GLH) [55]. The important residues for peptide engagement across eight class A GPCRs are marked by blue spheres. The peptide ligand ET-1 is shown as a cyan cylinder with two unstructured extended regions.

Similarly, we conducted the ΔΔG analysis on ECL2 residues and observed that all peptides interact favorably with the β-hairpin of this loop. Out of the three extracellular loops, ECL2 tends to be the most structured with a distinctive secondary structure of a twisted beta-hairpin conformation. In all structures of the nine complexes, ECL2 loops maintain the “open” conformation [68], opening a “gate” and allowing the peptide ligand to enter the core of the TM bundle from the extracellular region. Naturally, the peptides would interact with the β-hairpin of ECL2 β-hairpins at the “gate”, which connects the extracellular space to the inside transmembrane domain. This observation is also reflected in the computed ΔΔG of the interacting target residues. The ΔΔG analysis results suggest that the peptides generally engage with the β-hairpin of ECL2, especially at the tip where the three conserved residues (45.50, 45.51, and 45.52) are located (Figure 6 and Figure 7). 

### 2.4. A List of 14 Common Interacting Residues Suggests a General Peptide Recognition and Binding Mechanism among Nine Class A GPCRs

Despite a considerable diversity in size, sequence, secondary, and tertiary structure of the nine peptide ligands, we observed a significant overlap in the receptor region they bind to, particularly in a binding pocket between the outer leaflet portions of transmembrane helices (Figure 2). Using Rosetta [70,71], we calculated the per-residue ΔΔG of the interacting residues on the transmembrane helices, two conserved ECL1 residues (23.49 and 23.50), and three conserved ECL2 residues (45.50, 45.51, and 45.52). The details of the structure optimization and ΔΔG analysis protocols are listed in the Supplementary Material, and the ΔΔG values of all residues are listed in the Supplement Table S1. To make the optimization and ΔΔG analysis possible for the apelin/ApelinR (PDB ID: 5VBL) and the PMX53/C5aR (PDB ID: 6C1Q) structures, which contain a non-nature peptide backbone, we generated 5VBL* and 6C1Q* as natural-backbone peptide analogs of those structures. More specifically, the 5VBL* peptide ligand has the native apelin sequence, and the covalent bond between ornithine (ORN) at position 2 and the N-terminal acetyl group is omitted in the 6C1Q* peptide (Figure S3). Then, the GPCR residues were ranked based on their calculated ΔΔG. We selected 14 common residues with ΔΔG of less than −1 and contact peptide ligands in at least seven out of nine GPCR-peptide complexes. The details of the list and their locations on a GPCR structure are mapped in the structure of the ET-1/ETB receptor complex, as shown in Figure 6. This list of the top 14 residues implies a potential common peptide-binding mechanism among class A GPCRs. This common binding pocket encompasses two residues of TM2 (2.60 and 2.63), one from TM3 (3.32), three from TM6 (6.51, 6.55, and 6.58), five from TM7 (7.28, 7.32, 7.35, 7.36, and 7.39), and all three conserved ECL2 residues. More specifically, the common peptide engagement mechanism starts from the end of the β-hairpin of ECL2, extends to the tip of TM2, touches the extracellular half of TM7 and TM6, then ends at the core of TM3. The Supplementary Table S2 summarizes the non-Van Der Waal interactions between these 14 residues and the corresponding peptides. Although additional structures of peptide-GPCR complexes are still needed to validate our hypothesis of the common peptide binding pocket, this finding could help guide future structural studies of this family of GPCRs. 

**Figure 8:**
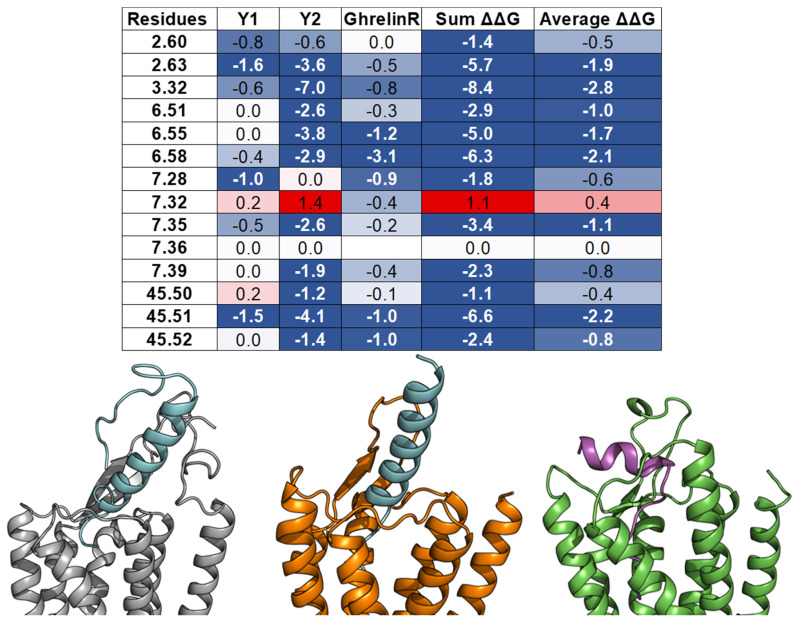
Models of peptide/class A GPCR complexes show that the peptides interact with the top 14 common residues. (From **left** to **right**): A table lists the ΔΔGs values of the 14 common residues of Y_1_ [56], Y_2_ [14], and ghrelin receptors [72], as well as their sum and average values. The absence of the ΔΔG values indicates that the corresponding residues do not interact with the peptide ligands. The residue ΔΔG cells are colored based on the ΔΔG values (negative: Blue, neutral: White, and positive: Red). The blank cells indicate that the residues do not interact with the peptide ligands. Models of NPY (cyan) bind with the Y_1_ receptor (grey) and the Y_2_ receptor (orange), and ghrelin (magenta) binds with the ghrelin receptor (green).

Herein, we examine whether the common binding mechanism agrees with the models of three class A GPCRs—Y_1_ [56], Y_2_ [14], and Ghrelin receptor [72]—and their endogenous peptide ligands—NPY and Ghrelin. In those studies, the peptide docking experiments were conducted using FlexPepDock [73] with constraints from mutagenesis, cross-linking, and NMR data. For each complex, the ΔΔG analysis was performed on an ensemble of docking models. The per-residue ΔΔG values were assigned to the interacting residues of the GPCR targets. The peptides’ binding pockets contain all of the 14 common residues, except for ghrelin, which does not contact the residue 7.36. Furthermore, most of the interactions between the common residues and NPY or ghrelin are favorable or at least neutral, except for the high ΔΔG value of residue 7.32 from Y_2_ (Figure 8). These results imply that the observation of the common peptide engagement pocket can also be applied to the docking study of peptide class A GPCRs, especially with limited experimental data.

A GPCR pharmacogenomics study has extracted polymorphism data for the coding-region of the 108 GPCR drug targets [74]. From the data provided by the authors, we found around 30 relevant GPCR mutants that were predicted to be deleterious by the sorting intolerant from tolerant (SIFT) [75] or Polyphen [76]. Those 30 genetic invariants have population allele frequencies of around 1 to 28 over 120,000 individuals and are related to the shared peptide interacting residues or are close to those residues. The table containing the information regarding the relevant mutants of peptide and protein binding class A GPCRs is summarized in the Supplementary Table Peptide_binding_pocket_genetic_variants.xlsx. The data suggest the great potential of the proposed common peptide-binding pocket as drug targets for class A GPCRs.

## 3. Structural Changes in Peptides Induced by Receptors Are Critical for Binding

This theme of conformational change in peptides in their bound state is not unique to peptide-GPCR recognition. Studies of ubiquitin by X-ray crystallography bound to various substrates identified several unique conformations. However, the NMR analysis revealed that all of these conformations existed simultaneously in the solution, demonstrating that the conformational selection drove the binding recognition event [77]. Peptide binding sites have been characterized to require unique conformations of peptide ligands in GPCRs [78], proteases [79], and other systems, including antibodies and a major histocompatibility complex [80]. To the best of our knowledge, there has not been a review of the conformational changes that the peptide ligand must undergo from their unbound to bound states at GPCRs. These changes have relevance in the future determination of structure and dynamics and thus in peptidomimetic drug discovery. The following section will highlight examples of peptide structural dynamics focusing on the conformational changes observed during the binding event.

### 3.1. Neurotensin

Neurotensin (NT) is a tridecapeptide [81] with the C-terminal six residues known as NT(8-13) responsible for receptor activation [82]. Original NMR studies of full-length NT in aqueous solution, methanol, and SDS (a membrane-mimic) found that under all conditions, the peptide was unstructured [83]. In contrast, significant chemical shift perturbations were observed for the C-terminal NT(8-13) upon binding to the receptor, indicating a conformational change when bound [84]. This structural rearrangement was subsequently confirmed by determining the structures of free, membrane-bound, and receptor-bound NT(8-13) with solid-state NMR [85] and molecular dynamics [86]. These studies found that both the solution and membrane-bound states contained no defined structure, while the receptor-bound peptide possessed an extended β-strand conformation. Knowledge of this extended binding pose allowed for the design of constrained peptides that reinforced the need for this conformation in the bound state. This further explained the reduced activity of end-to-end cyclization of NT(8-13) as it prevented the extended conformation [87].

### 3.2. Apelin

The apelin peptides are a family of peptides all formed from the same prohormone, but with subsequent N-terminal proteolytic processing. The structure-activity relationships (SAR) analysis on the peptide identified a primary binding motif of the last five C-terminal residues with a secondary binding motif located four residues away [88,89,90]. CD studies of the peptide revealed that in the solution, the apelin peptides possessed no structured regions [88,91]. The secondary structure could be induced by lowering the temperature of the solution [91] or the addition of membrane mimetics [91]. The regions that became ordered under these conditions were the same regions that were previously identified in SAR studies as the binding motifs. When the structure of APJR bound to an apelin mimetic was determined, it was found that the apelin mimetic adopted a conformation that allowed for an ordered presentation of these two binding motifs at distinct regions of the receptor [17]. Interestingly, the mutagenesis and MD simulations of apelin-13 in the crystal structure revealed that the native apelin peptide binds in a similar orientation as the crystalized ligand [17].

### 3.3. Endothelin

The endothelin peptides are a family of three 21-amino acid long peptides containing two internal disulfide bonds. Multiple NMR and X-ray studies have characterized the structure of these peptides to fold into a defined horseshoe orientation from residues 1 through 15 with residues 8–15 forming an α-helix [92,93,94,95,96,97]. This horseshoe orientation is stabilized due to the disulfide bonds and is lost when the disulfides are interrupted [22]. The C-terminus beyond residue 15 is highly dynamic, adopting helical structures [93] or extended structures [95,96] depending on the conditions of the experiment. In some cases, it is very poorly resolved that the structure could not be assigned to these residues [92,94]. However, the C-terminus is critical for the activity at ETA/B and should bind in an ordered pocket within the receptor [98]. As seen in the ET-1/ETB co-crystal structure, the overall conformation of ET-1 remained mostly unchanged from the solution since the two disulfide bonds reduced its structural flexibility [55]. However, the C-terminus of the ligand unwinds to bind within the receptor core, while remaining close to the ligand N-terminus. This orientation of the C-terminus with respect to the N-terminus is found in two of the 10 ensemble structures of a snake venom toxin with high sequence similarity and identical disulfide linkage as ET-1, suggesting that the peptide can sample this conformation, albeit at a low population, in the solution [95]. Interestingly, the receptor in the bound state folds its ECL2 and N-terminus over the ligand, explaining the extremely slow off-rates exhibited by these peptides in vivo [99,100]. This structure clearly demonstrates that conformational changes in both binding partners are needed for full binding activity.

### 3.4. The Complement System Peptide Ligand C5a

The complement system is a peptide-receptor system comprising of two ligands (C3a and C5a) and three receptors (C3aR, C5aR1, and C5aR2, previously known as GPR77). Both peptide ligands contain three conserved disulfide bonds that play a role in defining the overall helical bundle fold, which has been observed repeatedly by crystallography and NMR [101,102,103,104,105,106,107,108,109,110,111]. While the full peptide is necessary for the activation of the receptors, the C-terminal segment is the activation segment that binds at the receptor core [112]. This C-terminal segment adopts a variety of conformations depending on the studied condition and lacks any secondary structure. One NMR study measured the chemical shifts in the C-terminal residues to find an α-helix folding back onto the helix-bundle [111], an unlikely conformation in the active state as this peptide must be “presented” to the receptor for activation. Modeling the C-terminus of C5a in a C5aR homology model also suggested that the endogenous peptide possessed a dramatically different conformation in the solution than in the bound state [113]. In fact, this proposed binding mode was very similar to the bound conformation of the cyclic hexapeptide PMX53 [62]. The ligand formed a beta-hairpin to interact directly with ECL2 via backbone hydrogen bonding. It is now understood that the cyclization enforces the conformation of the backbone orientation to predefine the backbone geometry needed for the interaction with ECL2. Additional modeling studies have supported this extended conformation of C5a and derivative peptides [114,115].

### 3.5. Ghrelin

The ghrelin peptide is a 28 amino acid polypeptide with an octanoyl lipid modification at position Ser^3^ [20]. This peptide is the only known lipid-modified peptide hormone in the human body, and it has been found that this lipid modification is critical for receptor activation [20] [116,117,118]. Structure-function studies on ghrelin initially identified that the N-terminus of the peptide was critical for binding and activating the receptor via two main interactions: The positively charged amino head group and the hydrophobic octanoyl chain at Ser^3^ [118,119]. However, beyond these rules, little was known regarding the binding mode or conformation of ghrelin at its receptor. NMR and CD spectroscopy studies of the peptide in the solution agreed that the peptide was highly disordered in the aqueous state [120]. Increasing the hydrophobicity of the solution with organic solvents or detergents seemed to increase the helicity of the central portion of the peptide, while the termini remained highly flexible [121,122,123,124]. However, recent NMR data of the peptide bound to its receptor revealed that a helix is found in the central peptide, while the N-terminal binding portion converged to a well-defined extended structure [72,125].

### 3.6. Gonadotropin-Releasing Hormone

Gonadotropin-releasing hormone (GnRH) is a decapeptide consisting of pyroGlu-His-Trp-Ser-Tyr-Gly-Leu-Arg-Pro-Gly-NH2. The evolutionary analysis reveals that the first four residues, the central Gly6 residue, and the last two residues are highly conserved [126]. This pattern of conserved residues suggests a dual binding mode that requires both termini to come into close contact with the receptor. An extensive mutagenesis on both the peptide and receptor implies an inverted horseshoe binding motif for receptor activation [127]. NMR studies of this peptide in the solution failed to identify a single conformation [128,129,130,131]. However, peak sharpening increases in the presence of membranes, suggesting a reduction in conformational dynamics [128]. Computer simulations also revealed a broad population of conformations that could exist with many low energy states containing a β-turn conformation in residues 5–7 [132]. The conformations of Gly6 adopt states that are inaccessible to any other L-amino acid, but represent low energy conformations of D-amino acids [133]. Substitution of this residue with a D-amino acid enhances the likelihood of the β-turn, thereby prestabilizing the conformation for receptor binding. Interestingly, a Gly6 substitution with D-Trp can overcome the loss of binding in an Arg8 to Gln mutation [134]. GnRH analogs, including goserelin, nafarelin, triptorelin, leuprorelin, buserelin, histrelin, and deslorelin, are used to treat hormone-sensitive diseases, such as breast and prostate cancer [135] and often contain a D-amino acid substitution at position 6. NMR studies of nafarelin find that unlike GnRH, this peptide readily adopts a β-turn conformation in an aqueous solution [136]. Similar results were obtained in the NMR analysis of leuprorelin [137]. All of the described findings imply that GnRH needs to select a particular conformation in the unbound state to bind at the GnRH receptor.

### 3.7. Neuropeptide Y

The neuropeptide Y (NPY) system consists of three 36 amino acid peptide amides (NPY, PYY, and PP) and four receptors (Y_1_, Y_2_, Y_4_, Y_5_) with differing affinities for the various peptide/receptor combinations [31]. Initial studies to parse out the specific interactions of these peptides revealed that the C-terminal six residues were the primary binding and activation epitope within the NPY peptides [138]. An X-ray crystal structure of avian PP revealed a disordered N-terminus with an α-helix from residue 14–31 and a disordered C-terminus [139]. Solution NMR studies showed a different structure with the helix present through the C-terminal end of the peptide [140]. The recent mutagenesis and docking study also suggested that the C-terminus of PP needed to unwind to bind to the Y_4_ receptor [141]. Characterization of NPY in the membrane-bound state by NMR, CD, and EPR found the helix extending from residue 14 through the C-terminus [142,143]. It was not until the peptide was structurally characterized in its Y_2_ receptor-bound state that it became clear that the C-terminus, although helical in its membrane-bound state, must unwind into an extended conformation for binding at the receptor [14]. The conformational change of the C-terminus was also observed in a study of NPY binding at the Y_1_ receptor [56]. However, in this study, photo-crosslinking revealed that the N-terminus of NPY was interacting with ECL2 rather than the central helix. This alteration of the second binding site interaction resulted in a distinct binding orientation of NPY at two if its four receptors (Figure 8). Furthermore, studies will need to be pursued to contrast the binding mode of NPY at the remaining receptors in order to understand the complete basis of subtype selectivity.

### 3.8. Opioid Peptides

The opioid receptor family, comprising of δOR, µOR, κOR, and NOP, responds to various endogenous peptides, including endorphins, dynorphins, and enkephalins. These peptides contain a common N-terminal motif of YGGF followed by diverging residues. It is suggested that the N-terminal motif is the activation sequence, while the remaining residues confer receptor selectivity, the so-called “message-address” paradigm [144]. Once again, there is a conformational heterogeneity within the population of these peptides in both the aqueous- and membrane-bound states. A study of the peptide dynorphin B in the presence of the κOR found that the central portion of the peptide formed a well-defined α-helical turn, while the N- and C-terminal residues are structurally disordered [145]. It was interesting that multiple conformations were found for the N-terminal motif in the bound state. This conformation diversity contrasts with molecular dynamics simulations run on the DAMGO peptide bound in the µOR-G_i_ cryo-EM structure with the bound synthetic peptide [61]. Here, the researchers found that the peptide was relatively stable in its conformation over time within the binding pocket. At present, it is unclear if this conformational stability is due to the alterations of the peptide backbone in this synthetic peptide derivative, stabilization due to activation state or a difference between the binding pockets of µOR and κOR.

## 4. Implications for Future Studies

The flexibility of the peptide ligands and the extracellular loops of the receptor mandate the study of the structure and dynamics of peptide-activated GPCRs in tandem. X-ray crystallography and Cryo-EM will provide critical snapshots that display key structural determinants of peptide/receptor interactions. However, these studies need to be complemented by spectroscopic investigations that study the structure in the context of dynamics to gain a complete picture of the activation mechanism. While exciting progress in this area has been described over the past 5 years, we are only at the beginnings of these integrated approaches to study the structural dynamics of peptide-activated GPCRs. It is undeniable that interdisciplinary scientist teams are vital to the success of these studies, including experts in crystallography, spectroscopy, biochemistry, pharmacology, and modeling. Some of the computational technologies to integrate structural and dynamical data from various methods need to be optimized. However, since different methods introduce individual biases onto highly engineered systems, those systems need to be adequately considered when drawing conclusions for the wild-type ligand/receptor pair.

### 4.1. Peptides Need to Be Characterized in Their Bound State

**Figure 9:**
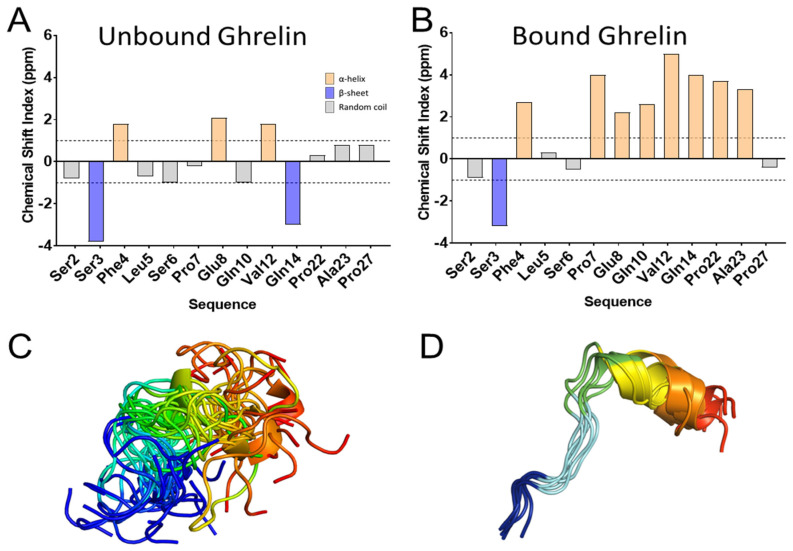
NMR measured conformational change in ghrelin upon the binding receptor. (**A**,**B**) Chemical shift index measurements of select residues in the ghrelin peptide in the presence of empty membrane or membrane containing ghrelin receptor [72,124]. These measurements identify a degree of secondary structure formation in the presence of receptor. (**C**,**D**) The chemical shifts were used to build models of ghrelin peptide in its two states, colored blue to red from N- to C-terminus.

Several peptide hormones have been examined to understand their structure via NMR or CD in solution. These include motilin [146], prolactin-releasing peptide [147,148], vasopressin [149], relaxin [150,151], and somatostatin analogues [152,153,154,155]. In contrast, relatively few examples of peptides exist, which are studied in both their solution and bound states. These include the peptides neurotensin, NPY, ghrelin, and bradykinin [14,32,56,72,85,124,142,156,157]. A common theme in all these studies and the ones mentioned above is that the conformations of the peptides in their unbound states are distinct from their bound state (Figure 9). This conformation differentiation is perhaps unsurprising as the individual degrees of freedom in each amino acid are high in a peptide. In contrast, the receptor binding pocket imposes a stringent constraint on the conformation of these peptides. This theme of conformational sampling is analogous to the change in extracellular loop conformations in C5aR when bound to a small molecule or peptide ligand [58,62]. Given these differences, it is necessary to study these peptides in the presence of their cognate receptors to develop a full understanding of the molecular basis of peptide recognition.

**Figure 10:**
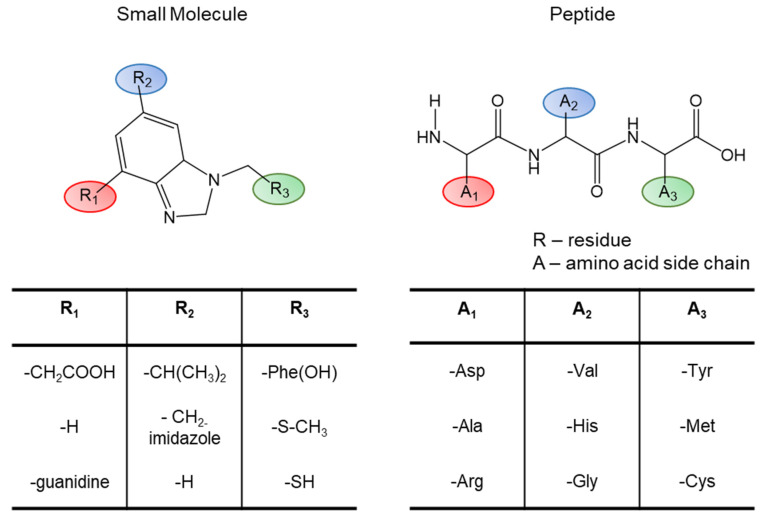
Schematic of peptide structure-activity relationships (SAR). In the same way as swapping chemical moieties for the small molecule (SAR). Peptide mutagenesis and alanine scanning are important tools for determining the peptide functionality at a given receptor.

Of note, the studies described in the above section rely on a variety of biophysical techniques for structural characterization. While X-ray crystallography and, in some cases, cryo-EM can reveal the conformations of peptides binding to GPCRs, this is currently rare. This lack of structure availability is likely due to the inherent flexibility of peptide ligands, as described, which can hinder the crystallization process or identification of class averages. Complementary to these techniques, several studies have utilized NMR and CD to characterize the peptide structure. CD provides readily accessible information to the overall secondary structure changes in varying environments. However, often the structural details can only be assessed qualitatively. In addition, providing residue-based structural data on the basis of CD measurements is impossible. In contrast, NMR can provide detailed information on a residue and atomic level regarding the structural properties of these peptides. To this end, specific ^13^C and/or ^15^N labeling of the peptide ligand is usually required, which is easily done using solid phase peptide synthesis. In this way, detailed structural information for an individual residue can be obtained as described above. In addition to structural data, NMR, especially using the saturation transfer difference (STD-) NMR, reveals information on population dynamics that may provide insights in the binding recognition process. Moreover, the transferred nuclear Overhauser effect (also called exchange-transferred NOE) method could be used to study the structural dynamics of the ligand from solution NMR. Additional techniques used in the studies as mentioned earlier include EPR, H/DX-MS, cross-linking, and molecular modeling. Finally, a powerful method used for decades in peptide ligand studies is the use of mutational analysis [158]. Alanine scanning and backbone modification of peptides is analogous to traditional SAR studies of small molecule ligands (Figure 10). Future studies will likely need to combine a multiple of these techniques in order to arrive at reliable understandings of these peptide-receptor complexes.

### 4.2. Mimetics of the Bound-State Conformations Can Aid in Structure Determination and Drug Discovery

As evidenced by the apelin, µOR, and neurotensin crystal structures, conformational stabilization or truncation of flexible components within the peptide ligands can assist in the crystallization of these complexes. While NT(8-13) and AMG3054 are less perturbed mimetics of the neurotensin and apelin, respectively, DAMGO represents a more dramatic change from the endogenous peptide ligand structure. Interpretation of these structures will need to be verified for the endogenous peptide ligands. SAR studies on peptides with no known crystal structures will be invaluable for understanding the conformational constraints required for these peptides in the bound states. Future crystallization trials with these conformationally constrained peptide derivatives will increase the likelihood of a stable crystal with interpretable density at the ligand binding site. Simultaneously, as the conformational constraints of these ligand binding sites become better understood, the development of more potent drug therapies may become more feasible. This vision has been evidenced clearly with the development of super-agonists for the gonadotropin-releasing hormone receptor. The addition of a D-amino acid enhanced the β-turn in the peptide that is needed for the bound state. It is suggested that the pre-orientation of the ligand conformation will reduce the entropic cost of binding, thereby increasing the affinity at the receptor. However, this suggestion has not yet been validated with stabilization attempts of neurotensin derivatives, in which the best derivatives are still only on par with the endogenous peptide. Therefore, this theme will need to be investigated in future drug developments to see if this consistently holds.

## 5. Conclusions

Peptide-binding GPCRs represent nearly a quarter of the druggable human GPCR superfamily. Our analysis discovered a common set of 14 residues that were shown to interact with peptide ligands among all of the available co-crystal structures. This shared binding site suggests a potential general pattern in peptide engagement among class A GPCRs. Additional studies of structure and dynamics may reveal how specific peptide-receptor recognition may formulate a general mechanism of activation for this family of receptors. Molecular dynamics simulation studies can be conducted to sample the energy landscape of the peptide activation mechanism [159]. Kinetics studies on binding between peptides and this class of receptor could also be applied to elucidate potential interactions that govern k_on_ and k_off_ of the indigenous peptide ligand engagement. This information will be essential for designing therapeutic modulators for peptide-binding class A GPCR [160]. Additionally, the wealth of ligand-GPCR interactions data available will enable deep learning models to be trained on and predict potential peptide-GPCR interactions or design novel potent biologic targeting GPCRs [161,162]. These common peptide-GPCR interactions could help guide the future exploration of the ensembles of protein-ligand conformations through computational modeling and various experimental techniques. The strength of computation lies in its ability to accurately use sparse experimental data to predict these types of interactions. Therefore, an iterative approach between computational sampling and energy minimization can be combined with restraints derived from a diversity of experimental methods. Incorporating a wide variety of complementary experimental techniques allows the integration of each method’s advantages in providing less ambiguous restraints: NMR provides dynamic restraints, X-ray provides rigid high-resolution restraints, and mutational studies and cross-linking allow single residue-specific restraints. These experimental restraints limit the search space of possible conformations, allowing for more accurate sampling in modeling. Then, these predictions can be used to guide the design of future experiments.

Furthermore, recent studies imply that the receptors influence the conformation of their peptide ligands, and that the peptide ligand can alter the conformation of the receptors’ extracellular loops. However, structural and dynamical studies on the peptide ligands or receptors are often pursued independently. Our current understanding suggests that conformational selection is a prime driver of receptor recognition. As a result, it is essential to study the receptor in the presence of cognate ligand and design experiments to define that interface, since we see that the conformation of a peptide in the absence of the receptor does not predict its conformation in the receptor-bound state. Moreover, the authors will likely define essential differences in these systems’ structural dynamics that evolved to allow for their diverse functions.

## Figures and Tables

**Table 1:** Examples of peptide hormone modifications.

Peptide Modification	Example	Function
C-terminal amidation	Neuropeptide Y (NPY), neuromedin B	C-terminal amidation reduces the overall charge of a peptide, forms key hydrogen interactions that are important for the potency of the peptide [15], and increases the metabolic stability of peptides as well as their ability to resist enzymatic degradation [16]
N-terminal pyroglutamic acid	Thyroid stimulating hormone (TSH), gonadotropin-releasing hormone I (GnRHI), regulated upon activation, Normal T cell expressed, and presumably secreted (RANTES)/chemokine ligand 5 (CCL5)	The pyroglutamic acid is often involved in peptide-receptor recognition and potency [17], and provides stability against N-terminal degradation [18]
Bromination	Neuropeptides B and W (NPBW)	Bromination on N-terminal tryptophan might protect the peptide from amino-peptidases’ degradation [19]
Lipidation	Ghrelin	The attached lipid group (e.g., octanoyl group) is essential to the activity of the peptide [19] and affects the hydrophobicity of the peptide [20]
Disulfide bridge formation	Endothelin, vasopressin	The disulfide bonds stabilize the defined secondary structure [21], stabilizing the bound conformation of the peptide [22]
Differential proteolysis	Bradykinin, angiotensin, NPY/NPY3-36, apelin (Ape)-13/Ape-17/Ape 36, adrenocorticotropic hormone (ACTH), pro-opiomelanocortin (POMC) cleavage yielding α-, β-, and γ- melanocyte-stimulating hormone (MSH), and endorphins	Proteolysis can switch the activity of the peptides on and off [23] or differentiates the binding selectivity and the biological responses of the peptides [24]

## Supplementary Materials

The following supporting information can be downloaded online. Table S1: Per-residue ΔΔG values for TMs and conversed loop residues of nine class A GPCR structures; Table S2: Non Van-der Waals interactions between the set of 14 common interacting residues of different peptide-bound class A GPCRs to their corresponding peptides; Figure S1: Per-residue ΔΔG values for TMs and conversed loop residues of nine class A GPCR structures; Figure S2: Per-residue ΔΔG values for TMs and conversed loop residues of nine class A GPCR structures that are sorted in ascending order; Figure S3: Comparison between the PMX53 structures in the Rosetta relaxed model (pink) and in the crystal structure (PDB ID: 6C1Q) (cyan). While the sidechain of ornithine (ORN) at position 2 and the carboxylate group of ARG at position 6 are linked in the crystal structures, they form a salt bridge in the Rosetta relaxed models.
