# Supplementary material for: The Structural Basis of Peptide Binding at Class A G Protein-Coupled Receptors
Source: Molecules. 2021 Dec 30;27(1):210. doi: 10.3390/molecules27010210 (PMC8746363; doi:10.3390/molecules27010210)
Supplement: Supplementary file 1 [file molecules-27-00210-s001.zip › molecules-1078932-supplementary/molecules-1078932-supplementary.pdf]

molecules

MDPI

Table S1. List, description, and weights of the two-body scoring terms used in calculating ΔΔG. For the mathematical models and physical concepts that underlie those scoring terms, please consult the reference paper [1].

Two-body terms	Description	weight
fa_atr	Attractive energy between two atoms on different residues seperated by a distance d	1
fa_rep	Repulsive energy between two atoms on different residues seperated by a distance d	0.55
fa_elec	Energy of interaction between two nonbonded charged atoms seperated by a distance d	1
fa_sol	Gaussian exclusion implicit solvation energy between protein atoms in different residues	1
dslf_fa13	Energy of disulfide bridges	1.25
Hbond_lr_bb	Energy of long-range hydrogen bonds	1
hbond_sr_bb	Energy of short-range hydrogen bonds	1
hbond_bb_sc	Energy of backbone-side-chain hydrogen bonds	1
hbond_sc	Energy of sidechain-sidechain hydrogen bonds	1

Table S2. Non Van-der Waals interactions between the set of 14 common interacting residues of different peptide-bound class A GPCRs to their corresponding peptides. The residue list is sorted in their ascending average ΔΔG order (see Figure 7). The interactions analysis was performed using MOE [2]. Types of interactions: (H): hydrogen bond; (I): ionic interactions; (IH): saltbridge; (A): interaction that involve aromatic pi bonds; (C): disulfide bonds. The residues are numbered according on the Ballesteros-Weinsein numbering scheme [3]. For each residue position, the values of the total and average number of non-Van Der Waals interactions are colored in green such that darker hues signify higher values. The absence of the interaction values means the residue do not form non-Van der Waals interactions with the peptide ligands.

Receptor<br>Residue #	CXCR4<br>4RWS	ETBR<br>5GLH	CCR5<br>5UIW	apelinR<br>5VBL	US28<br>5WB2	C5a1R<br>6C1Q	MuOR<br>6DDF	CCR5<br>6MEO	AT1R<br>6OS0	Total number of interactions	Average number of interactions
7.39	H,H		H		H			I		5	0.56
2.60	A	H								2	0.22
45.51	C		H							2	0.22
45.52		H,H	H,H			H,H		H	H	8	0.89
7.32			IH		H,H				IH,H	5	0.56
7.35						H,IH		H	A	4	0.44
6.58	H,IH	H,H,H	H	H			H	H	IH	10	1.11
6.51				H	H	H		IH		4	0.44
2.63	IH,H	H,H,H						A		6	0.67
3.32		H					IH			2	0.22
7.36		H	H						A	3	0.33
1.24	H,H		H		H					4	0.44
45.50	H,H					H				3	0.33
7.28	I	A		I						3	0.33
6.55		IH,IH		IH						3	0.33

Figure S1. Per-residue ΔΔG values for TMs and conversed loop residues of nine class A GPCR structures.

Receptor	CXCR4	ETBR	CCR5	apelinR	US28	C5a1R	MuOR	CCR5	AT1R
**Residue #**	**4RWS**	**5GLH**	**5UIW**	**5VBL***	**5WB2**	**6C1Q***	**6DDF**	**6MEO**	**6OS0**
**1.22**		-0.03							
**1.23**		-0.54							
**1.24**	-4.19	-2.31	-2.08		-4.88			-0.39	
**1.25**	-0.40	-1.90	0.00		-0.95			-0.03	
**1.26**		-0.03						-0.51	
**1.27**	-0.02	-1.66	-1.15					-1.72	
**1.28**			-1.61		-4.56			-2.55	
**1.29**			0.00		-0.01	0.02		0.00	
**1.30**								0.00	
**1.31**	0.01		-1.27		-0.03			-0.68	0.00
**1.32**			-0.01		-0.93	-0.04		-0.10	
**1.33**					0.00				
**1.35**			-1.70		-0.58	0.00		-0.27	0.00
**1.36**					0.00				
**1.39**	0.00		-0.62	0.00	-0.40	-0.40	0.00	-0.05	-0.24
**23.49**		-0.56		-0.60		0.00		0.01	
**23.50**	-1.15	-1.33				-0.41	-1.24	-0.06	
**2.53**		-0.18	0.00	-0.01	0.01			0.00	0.00
**2.56**	-0.05	-0.01	0.00				-0.15	0.00	
**2.57**					0.00	0.00			0.00
**2.59**	-1.13	-1.85	0.00			0.00	-0.22		
**2.60**	-5.43	-4.47	-3.76	-0.23	-2.36	-2.26	-1.68	-2.76	-3.14
**2.61**	-0.01	0.00	-0.04						0.00
**2.62**							0.00	0.00	
**2.63**	-6.48	-3.62	-2.67	-0.18	-0.40	-0.24	0.12	-1.87	-0.58
**2.64**	-0.60	-0.08	-1.60	-0.12	-3.11	-0.63	0.00	-0.25	0.00
**2.65**	0.00		0.00	-1.80				-0.02	
**2.66**		0.00		-2.72				0.07	
**2.67**				-2.32		0.00			
**3.24**	0.00	-0.03							
**3.25**	-0.13	-0.08	-0.19		-0.01	0.00	-0.19	-0.05	
**3.26**		0.00							
**3.28**	-1.83	-1.34				-0.06	-1.04		0.00
**3.29**	-0.82	-1.08	-0.64	-0.02	-0.05	-3.10	-2.43	-0.33	0.02
**3.30**						-0.01			
**3.31**						0.02			
**3.32**	0.00	-1.24	-1.98	-1.38	-1.20	-0.48	-5.70	-1.77	-1.87
**3.33**		-1.18	-1.63	-0.89	-0.51	0.00	-1.53	-1.89	-0.91
**3.34**		0.00							-0.01
**3.35**		0.00							
**3.36**		-0.64		-1.15	-0.86		-1.83		-2.24
**3.37**							0.00		-0.70
**45.50**	-3.45	-0.32	-0.61	-0.14	-1.23	-4.15	-1.55	-0.72	0.35
**45.51**	-2.18	-4.91	-3.04	-0.22	-2.38	-3.04	-2.73	-1.91	-3.36
**45.52**	0.00	-2.15	-4.13	-0.28	0.07	-5.39	-0.38	-4.06	-5.28
**4.56**									-0.01
**4.57**									-0.01
**4.59**								-0.06	0.00
**4.60**		0.65	-0.09		0.00	0.02		-0.65	-0.06
**4.61**								-0.01	
**4.62**								0.00	
**4.63**							0.00	0.00	
**4.64**		-0.27	-0.81	-0.17		0.00			-2.00

Receptor	CXCR4	ETBR	CCR5	apelinR	US28	C5a1R	MuOR	CCR5	AT1R
**Residue #**	**4RWS**	**5GLH**	**5UIW**	**5VBL***	**5WB2**	**6C1Q***	**6DDF**	**6MEO**	**6OS0**
**5.31**			-5.85	-0.04		0.00		-3.45	
**5.32**			-0.12	-0.43		0.13		0.00	
**5.34**		-0.03	-0.03		0.00			-1.03	0.00
**5.35**		-1.06	-2.93	-1.45	-0.24	-2.02		-1.47	
**5.36**		-0.21	0.00		0.00	0.00		0.00	
**5.37**		0.00							
**5.38**		-3.44	-0.73		-1.14	0.00		-1.62	-0.11
**5.39**		-0.17	-0.29		-1.12	-0.07	-0.02	0.02	
**5.42**		-1.03	-0.98		-0.69	0.17	-0.65	-0.50	-3.98
**5.43**		0.00					0.01		
**5.46**		-0.04					-0.09		0.01
**5.47**							0.00		
**6.48**		-0.98		0.09	-0.38		-0.37	0.00	-0.25
**6.50**						-0.01			
**6.51**	0.12	-2.82	0.03	-5.86	-3.84	-0.81	-3.10	-0.21	-0.13
**6.52**		-0.24			0.00	-0.02	-1.56		0.00
**6.53**						0.00	-0.01		
**6.54**		0.00	0.00	-0.91		-0.50	-0.03		
**6.55**	0.00	-2.09	-0.38	-4.27	-1.16	0.11	-1.91	-0.86	0.02
**6.56**						-0.02	0.01	0.00	
**6.57**				-0.02					
**6.58**	-4.68	-2.75	-0.91	-5.03	0.10	-1.64	-0.63	-1.67	-3.41
**6.59**		-0.06	-0.09	-0.07	0.00	0.04		-0.76	
**6.60**				0.01				0.00	
**6.61**	-0.21		-0.38	0.19	0.13			0.05	-1.02
**6.62**	-0.20	-2.68	0.20	-0.10	-0.48				-0.48
**6.63**				0.01					0.00
**6.64**	-0.06				-1.51				
**7.24**	-0.79	-0.98	-2.27	0.00	0.01				
**7.25**	-0.87	-0.38	-1.15	-0.01	-0.01				0.00
**7.26**	0.00								
**7.27**		-0.04							
**7.28**	-1.69	-4.26	-0.56	-3.13	-0.59	-0.05			-0.64
**7.29**	-0.03	-0.09	-0.01					-0.02	-0.01
**7.30**		0.01							
**7.31**	-0.08	-1.24	0.28	-0.81	0.00	-0.01		0.00	-0.02
**7.32**	-2.80	-6.23	-3.28	-0.78	-2.46	0.11		-0.60	-5.99
**7.33**		0.00	-0.04	0.00				-0.32	-0.05
**7.34**				0.00		-0.02		0.00	
**7.35**	-2.22	-0.04	-1.08	-5.61	-3.11	-5.42	-0.98	-1.92	-1.79
**7.36**	-0.81	-4.82	-2.78	0.01	-3.35	-0.69	0.00	-2.64	-0.23
**7.37**	0.00	0.00			0.00	0.00		0.00	
**7.38**		0.00		-0.03	-0.05	0.30			
**7.39**	-4.00	-3.73	-5.08	-1.29	-4.99	-3.27	-1.44	-0.60	-2.65
**7.40**		0.00	-0.01	0.00	-0.01	0.00			
**7.42**		-0.08		-0.26	-0.30		0.00		0.00
**7.43**	0.01	0.00	-0.79	-1.15	-1.23	-0.30	0.02	-0.05	-0.12

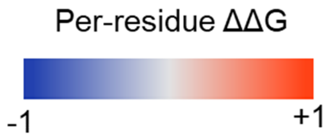

Figure S2. Per-residue ΔΔG values for TMs and conversed loop residues of nine class A GPCR structures that are sorted in ascending order.

CXCR4 4RWS	ETBR 5GLH	CCR5 5UIW	apelinR 5VBL*	US28 5WB2	C5a1R 6C1Q*	MuOR 6DDF	CCR5 6MEO	AT1R 6OS0	Average ΔΔG
-4.0	-3.7	-5.1	-1.3	-5.0	-3.3	-1.4	-0.6	-2.7	-3.0
-5.4	-4.5	-3.8	-0.2	-2.4	-2.3	-1.7	-2.8	-3.1	-2.9
-2.2	-4.9	-3.0	-0.2	-2.4	-3.0	-2.7	-1.9	-3.4	-2.6
-2.2	0.0	-1.1	-5.6	-3.1	-5.4	-1.0	-1.9	-1.8	-2.5
-2.8	-6.2	-3.3	-0.8	-2.5	0.1		-0.6	-6.0	-2.4
0.0	-2.2	-4.1	-0.3	0.1	-5.4	-0.4	-4.1	-5.3	-2.4
-4.7	-2.8	-0.9	-5.0	0.1	-1.6	-0.6	-1.7	-3.4	-2.3
0.1	-2.8	0.0	-5.9	-3.8	-0.8	-3.1	-0.2	-0.1	-1.8
-6.5	-3.6	-2.7	-0.2	-0.4	-0.2	0.1	-1.9	-0.6	-1.8
0.0	-1.2	-2.0	-1.4	-1.2	-0.5	-5.7	-1.8	-1.9	-1.7
-0.8	-4.8	-2.8	0.0	-3.4	-0.7	0.0	-2.6	-0.2	-1.7
-4.2	-2.3	-2.1		-4.9			-0.4		-1.5
-3.5	-0.3	-0.6	-0.1	-1.2	-4.2	-1.6	-0.7	0.4	-1.3
-1.7	-4.3	-0.6	-3.1	-0.6	-0.1			-0.6	-1.2
0.0	-2.1	-0.4	-4.3	-1.2	0.1	-1.9	-0.9	0.0	-1.2
		-5.9	0.0		0.0		-3.5		-1.0
	-1.1	-2.9	-1.5	-0.2	-2.0		-1.5		-1.0
		-1.6		-4.6			-2.6		-1.0
	-1.2	-1.6	-0.9	-0.5	0.0	-1.5	-1.9	-0.9	-0.9
-0.8	-1.1	-0.6	0.0	-0.1	-3.1	-2.4	-0.3	0.0	-0.9
	-1.0	-1.0		-0.7	0.2	-0.7	-0.5	-4.0	-0.9
	-3.4	-0.7		-1.1	0.0		-1.6	-0.1	-0.8
	-0.6		-1.2	-0.9		-1.8		-2.2	-0.7
-0.6	-0.1	-1.6	-0.1	-3.1	-0.6	0.0	-0.3	0.0	-0.7
0.0	-1.7	-1.2					-1.7		-0.5
-1.8	-1.3				-0.1	-1.0		0.0	-0.5
-1.2	-1.3				-0.4	-1.2	-0.1		-0.5
-0.8	-1.0	-2.3	0.0	0.0					-0.4
-0.2	-2.7	0.2	-0.1	-0.5				-0.5	-0.4
0.0	0.0	-0.8	-1.2	-1.2	-0.3	0.0	-0.1	-0.1	-0.4
-0.4	-1.9	0.0		-1.0			0.0		-0.4
	-0.3	-0.8	-0.2		0.0			-2.0	-0.4
-1.1	-1.9	0.0			0.0	-0.2			-0.4
	0.0		-2.7				0.1		-0.3
		-1.7		-0.6	0.0		-0.3	0.0	-0.3
-0.9	-0.4	-1.2	0.0	0.0				0.0	-0.3
			-2.3		0.0				-0.3
0.0		-1.3		0.0			-0.7	0.0	-0.2
	-1.0		0.1	-0.4		-0.4	0.0	-0.3	-0.2
-0.1	-1.2	0.3	-0.8	0.0	0.0		0.0	0.0	-0.2
0.0		0.0	-1.8				0.0		-0.2
	-0.2			0.0	0.0	-1.6		0.0	-0.2
0.0		-0.6	0.0	-0.4	-0.4	0.0	-0.1	-0.2	-0.2
	-0.2	-0.3		-1.1	-0.1	0.0	0.0		-0.2
-0.1				-1.5					-0.2
	0.0	0.0	-0.9		-0.5	0.0			-0.2
-0.2		-0.4	0.2	0.1			0.1	-1.0	-0.1
	-0.6		-0.6		0.0		0.0		-0.1
	0.0	0.0		0.0			-1.0	0.0	-0.1
		0.0		-0.9	0.0		-0.1		-0.1
	-0.1	-0.1	-0.1	0.0	0.0		-0.8		-0.1
						0.0		-0.7	-0.1
-0.1	-0.1	-0.2		0.0	0.0	-0.2	-0.1		-0.1
	-0.1		-0.3	-0.3		0.0		0.0	-0.1
	-0.5								-0.1

Receptor Residue #	CXCR4 4RWS	ETBR 5GLH	CCR5 5UIW	apelinR 5VBL*	US28 5WB2	C5a1R 6C1Q*	MuOR 6DDF	CCR5 6MEO	AT1R 6OS0	Average ΔΔG
**1.26**		-0.03						-0.51		-0.06
**5.32**			-0.12	-0.43		0.13		0.00		-0.05
**7.33**		0.00	-0.04	0.00				-0.32	-0.05	-0.05
**2.56**	-0.05	-0.01	0.00				-0.15	0.00		-0.02
**5.36**		-0.21	0.00		0.00	0.00		0.00		-0.02
**2.53**		-0.18	0.00	-0.01	0.01			0.00	0.00	-0.02
**7.29**	-0.03	-0.09	-0.01					-0.02	-0.01	-0.02
**4.60**		0.65	-0.09		0.00	0.02		-0.65	-0.06	-0.01
**5.46**		-0.04					-0.09		0.01	-0.01
**4.59**								-0.06	0.00	-0.01
**2.61**	-0.01	0.00	-0.04						0.00	-0.01
**7.27**		-0.04								0.00
**1.22**		-0.03								0.00
**3.24**	0.00	-0.03								0.00
**6.57**				-0.02						0.00
**7.34**				0.00		-0.02		0.00		0.00
**7.40**		0.00	-0.01	0.00	-0.01	0.00				0.00
**3.30**						-0.01				0.00
**3.34**		0.00							-0.01	0.00
**4.56**									-0.01	0.00
**4.57**									-0.01	0.00
**4.61**								-0.01		0.00
**6.50**						-0.01				0.00
**6.53**						0.00	-0.01			0.00
**6.56**						-0.02	0.01	0.00		0.00
**1.30**								0.00		0.00
**1.33**					0.00					0.00
**1.36**					0.00					0.00
**2.57**					0.00	0.00			0.00	0.00
**2.62**							0.00	0.00		0.00
**3.26**		0.00								0.00
**3.35**		0.00								0.00
**4.62**								0.00		0.00
**4.63**							0.00	0.00		0.00
**5.37**		0.00								0.00
**5.47**							0.00			0.00
**7.26**	0.00									0.00
**7.37**	0.00	0.00			0.00	0.00		0.00		0.00
**1.29**			0.00		-0.01	0.02		0.00		0.00
**5.43**		0.00					0.01			0.00
**6.60**				0.01				0.00		0.00
**6.63**				0.01					0.00	0.00
**7.30**		0.01								0.00
**3.31**						0.02				0.00
**7.38**		0.00		-0.03	-0.05	0.30				0.02

Per-residue ΔΔG

-1 +1

Figure S3. Comparison between the PMX53 structures in the Rosetta relaxed model (pink) and in the crystal structure (PDB ID: 6C1Q) (cyan). While the sidechain of ornithine (ORN) at position 2 and the carboxylate group of ARG at position 6 are linked in the crystal structures, they form a salt bridge in the Rosetta relaxed models.

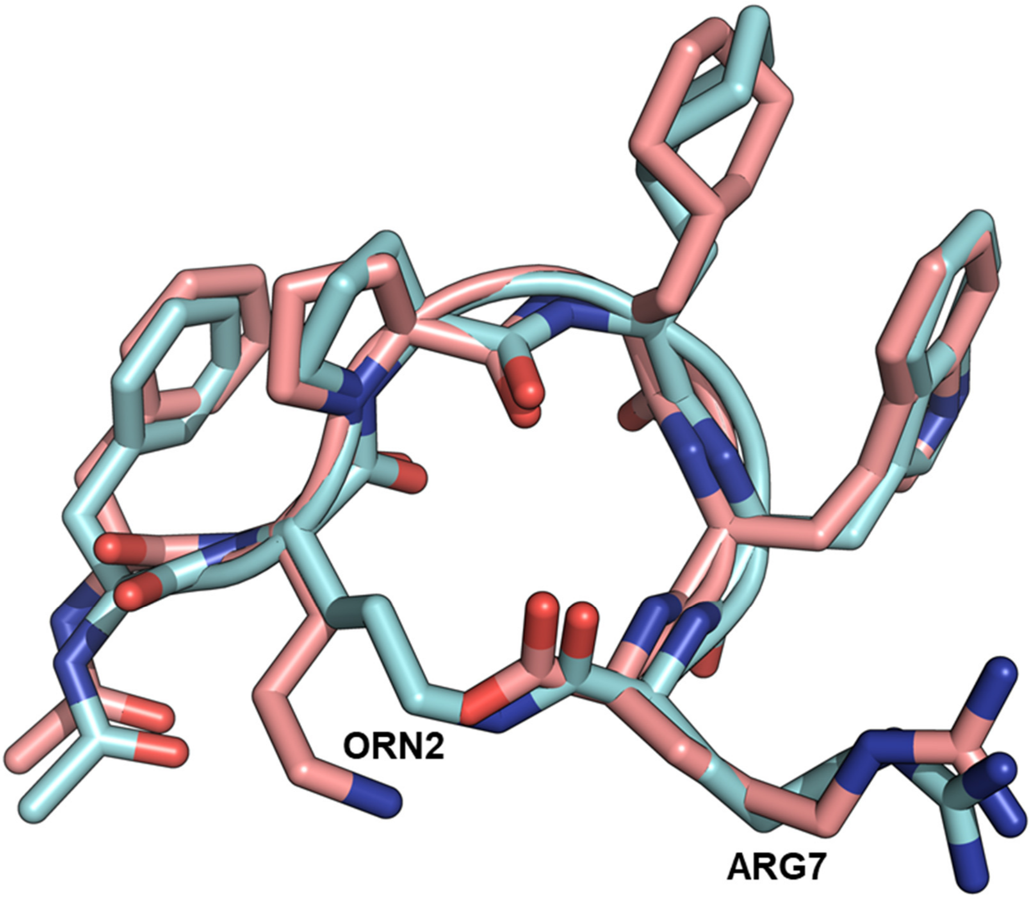
ORN2
ARG7

Methods

1. *Structure preparation:*

Nine structures are downloaded and the coordinates of the GPCR targets and the peptide ligands were extracted. The complex structures were then minimized by Rosetta backrub applied to the interface residues [4] followed by two cycles of fast relax [5]. Backrub movement mimics the backbone fluctuations observed in the crystal lattice [4]. The BackrubDD mover combined backrub movements with metropolis Monte Carlo to sample low energy backbone conformation that were close to the starting crystal structures in the context of the Rosetta all-atom force field. The FastRelax protocol found low-energy backbone and side-chain conformations near a starting conformation by applying ten repeats of five rounds of packing and minimizing, with the repulsive weight in the scoring function gradually increased from a very low value to the normal value from one round to the next [6]. For each complex, ten optimized model were generated. The sequence numbering table for each GPCR class A were extracted from GPCRdb database [7].

2. *Incorporate NCAAs into peptide modeling for 6C1Q*

A rotamer library of 1000 conformers of 3-cyclohexyl-L-alanine were generated using BCL:Conf [8, 9]. We used ornithine rotamer library that was generated in a previous study [10]. Those non-natural amino acids were then incorporated into the structural optimization step of the complex.

3. *Modeling of native peptide of 5VBL*

Since the structure of the ligand in the structure is derived from the C-terminus of apelin [11], we generated the model of the native apelin peptide and the apelin receptor using fix backbone design application in Rosetta [12] before the structural optimization step.

4. *ΔΔG analysis:*

Residue-pair Rosetta interaction energy between GPCR targets and peptide ligands were computed. For each model of the optimized structure ensemble, the ΔΔG value of each interaction target residue is the sum of computed pairwise interaction energy. The ΔΔG values of each residue were then averaged over 10 structures.

The protocol capture and code have been uploaded to github (link: https://github.com/vuoanh/peptide_binding_classA_GPCR_protocol_capture.git)

References

1. Alford, R. F.; Leaver-Fay, A.; Jeliazkov, J. R.; O'Meara, M. J.; DiMaio, F. P.; Park, H.; Shapovalov, M. V.; Renfrew, P. D.; Mulligan, V. K.; Kappel, K.; Labonte, J. W.; Pacella, M. S.; Bonneau, R.; Bradley, P.; Dunbrack, R. L.; Das, R.; Baker, D.; Kuhlman, B.; Kortemme, T.; Gray, J. J., The Rosetta All-Atom Energy Function for Macromolecular Modeling and Design. *J. Chem. Theory Comput.* **2017,** 13, (6), 3031-3048.
2. Vilar, S.; Cozza, G.; Moro, S., Medicinal chemistry and the molecular operating environment (MOE): application of QSAR and molecular docking to drug discovery. *Curr. Top. Med. Chem.* **2008,** 8, (18), 1555-72.
3. Isberg, V.; de Graaf, C.; Bortolato, A.; Cherezov, V.; Katritch, V.; Marshall, F. H.; Mordalski, S.; Pin, J.-P.; Stevens, R. C.; Vriend, G.; Gloriam, D. E., Generic GPCR residue numbers - aligning topology maps while minding the gaps. *Trends Pharmacol. Sci.* **2015,** 36, (1), 22-31.
4. Smith, C. A.; Kortemme, T., Backrub-like backbone simulation recapitulates natural protein conformational variability and improves mutant side-chain prediction. *J. Mol. Biol.* **2008,** 380, (4), 742-756.
5. Conway, P.; Tyka, M. D.; DiMaio, F.; Konerding, D. E.; Baker, D., Relaxation of backbone bond geometry improves protein energy landscape modeling. *Protein Sci.* **2014,** 23, (1), 47-55.
6. Nivón, L. G.; Moretti, R.; Baker, D., A Pareto-Optimal Refinement Method for Protein Design Scaffolds. *PLOS ONE* **2013,** 8, (4), e59004.
7. Isberg, V.; Mordalski, S.; Munk, C.; Rataj, K.; Harpsoe, K.; Hauser, A. S.; Vroling, B.; Bojarski, A. J.; Vriend, G.; Gloriam, D. E., GPCRdb: an information system for G protein-coupled receptors. *Nucleic Acids Res.* **2017,** 45, (5), 2936.
8. Mendenhall, J.; Brown, B. P.; Kothiwale, S.; Meiler, J., BCL::Conf: Improved Open-Source Knowledge-Based Conformation Sampling Using the Crystallography Open Database. *J. Chem. Inf. Model.* **2021,** 61, (1), 189-201.
9. Kothiwale, S.; Mendenhall, J. L.; Meiler, J., BCL::Conf: small molecule conformational sampling using a knowledge based rotamer library. *J. Cheminform.* **2015,** 7, 47-47.
10. Renfrew, P. D.; Choi, E. J.; Bonneau, R.; Kuhlman, B., Incorporation of noncanonical amino acids into Rosetta and use in computational protein-peptide interface design. *PloS One* **2012,** 7, (3), e32637-e32637.
11. Ma, Y.; Yue, Y.; Ma, Y.; Zhang, Q.; Zhou, Q.; Song, Y.; Shen, Y.; Li, X.; Ma, X.; Li, C.; Hanson, M. A.; Han, G. W.; Sickmier, E. A.; Swaminath, G.; Zhao, S.; Stevens, R. C.; Hu, L. A.; Zhong, W.; Zhang, M.; Xu, F., Structural Basis for Apelin Control of the Human Apelin Receptor. *Structure* **2017,** 25, (6), 858-866 e4.
12. Kuhlman, B.; Dantas, G.; Ireton, G. C.; Varani, G.; Stoddard, B. L.; Baker, D., Design of a Novel Globular Protein Fold with Atomic-Level Accuracy. *Science* **2003,** 302, (5649), 1364.
